# Clinical features and oncologic outcomes of primary retroperitoneal ganglioneuroma: a retrospective cohort study of 51 patients from a high-volume sarcoma center

**DOI:** 10.3389/fsurg.2025.1701114

**Published:** 2025-11-11

**Authors:** Wenjie Li, Mengmeng Xiao, Chengli Miao, Boyuan Zou, Shibo Liu, Mei Huang, Haicheng Gao

**Affiliations:** 1 Department of Retroperitoneal Tumor Surgery, Peking University International Hospital, Beijing, China; 2 Department of General Surgery, Peking University People’s Hospital, Beijing, China

**Keywords:** ganglioneuroma, clinicopathological feature, outcome, complications, surgical treatment

## Abstract

**Background:**

Retroperitoneal ganglioneuroma (RGN) is a rare, benign tumor derived from neural crest cells of the sympathetic nervous system. Due to its rarity and complex management, clinical understanding remains limited. This study aimed to analyze the clinical features and surgical outcomes of RGN.

**Methods:**

A retrospective analysis was performed utilizing the retroperitoneal tumor database of Peking University International Hospital. Patients who underwent surgical resection for pathologically confirmed primary RGN between January 2015 and August 2024 were included. Systematic postoperative follow-up was conducted to assess outcomes.

**Results:**

Fifty-one consecutive patients (18 males, 33 females; median age 28 years, range 12–73) with newly diagnosed RGN were enrolled. Clinical presentations were heterogeneous: 29 cases (56.9%) were incidental findings on physical examination, 16 (31.4%) reported abdominal discomfort, 3 (5.9%) had lumbago, and single cases presented with hematuria, chest tightness, or lower limb pain. R0/R1 resection was achieved in 45 patients (88.2%). Postoperative complications occurred in 11 patients (21.6%), including gastroparesis (*n* = 3), pancreatic fistula (*n* = 3), liver function impairment (*n* = 2), wound infection (*n* = 2), and one mortality due to intestinal ischemia and necrosis. Histopathology confirmed GN in all cases. At median follow-up of 62 months (90.2% follow-up rate), no recurrences, metastases, or disease-specific deaths occurred—including in R2 resection patients (*n* = 6).

**Conclusions:**

Retroperitoneal ganglioneuroma (RGN) is a rare benign tumor that frequently presents with nonspecific symptoms. While surgical resection remains the cornerstone of management, it is best undertaken at specialized, high-volume sarcoma centers to mitigate operative risks. When complete excision is precluded by critical vascular involvement, subtotal resection represents a judicious alternative. For selected patients with small, asymptomatic tumors—particularly those with elevated surgical risk—active surveillance is a reasonable option. Despite a generally favorable prognosis, long-term follow-up is recommended.

## 1 Introduction

Retroperitoneal ganglioneuroma (RGN) is a rare, benign neurogenic tumor originating from neural crest cells, characterized as the most differentiated form of peripheral neuroblastic tumor (1). Although it can occur across all age groups, RGN demonstrates a predilection for children and young adults. The retroperitoneum (accounting for 32%–52% of cases) and posterior mediastinum (39%–43%) represent the predominant anatomical sites (2). Patients typically remain asymptomatic until tumor progression induces compression of adjacent organs or vascular structures (3, 4). Owing to its rarity, RGN commonly leads to diagnostic delays or misdiagnosis. Complete surgical resection presents substantial technical challenges, particularly when the tumor envelops major retroperitoneal vasculature (e.g., aorta, inferior vena cava) (5). While RGN is characteristically benign, rare instances of malignant transformation to ganglioneuroblastoma or neuroblastoma have been documented (6, 7). This retrospective study of 51 patients with primary RGN provides valuable insights into the clinical management and oncological outcomes of this rare neurogenic tumor.

## 2 Patients and methods

### 2.1 Patient selection

A retrospective review was conducted of all consecutive patients undergoing surgical resection for RGN at Peking University International Hospital between January 2015 and August 2024. Inclusion criteria comprised: first-time surgical resection and histopathological confirmation of primary RGN. Exclusion criteria included: recurrent tumors or history of other primary malignancies. Ultimately, 51 patients with RGN were enrolled in the final cohort analysis.

### 2.2 Data collection

Data were extracted from our prospectively maintained retroperitoneal tumor database, encompassing demographic characteristics, clinical manifestations, preoperative laboratory and imaging findings, operative details, and pathological features. Systematic post-operative follow-up was conducted via telephone interviews or clinical visits. The final follow-up date was February 1, 2025. The composite primary endpoint comprised disease-specific mortality or histologically/radiologically confirmed recurrence. Survival analysis was performed using these pre-defined endpoint events during follow-up as the evaluation basis.

### 2.3 Statistical analysis

Categorical variables are presented as frequencies and percentages (%). Continuous variables are expressed as mean ± standard deviation (SD) or median with interquartile range (IQR), as appropriate. Follow-up duration and survival times were right-censored at the final follow-up date (February 1, 2025) for patients who remained alive without recurrence or were lost to follow-up. Recurrence-free survival (RFS) was estimated using the Kaplan–Meier method. Statistical significance was defined by a two-sided *P* value <0.05. All analyses were performed using IBM SPSS Statistics, Version 22.0 (IBM Corp., Armonk, NY, USA).

### 2.4 Ethics approval and consent to participate

The study protocol received approval from the Institutional Review Board (IRB) of Peking University International Hospital. Written informed consent was obtained from all participating patients prior to inclusion. For participants with impaired decision-making capacity, consent was provided by legally authorized representatives. All study procedures adhered strictly to the ethical principles established by: the institutional and national research ethics committees and the 1964 Declaration of Helsinki and its later amendments (currently the 2013 Fortaleza version).

## 3 Results

### 3.1 Demographics and clinical manifestations

This study enrolled 51 consecutive patients (male:female ratio 18:33; 35.3% male). The median age was 28 years (range: 12–73). Clinical manifestations varied considerably among patients. Key demographic characteristics (age, sex, comorbidities) are comprehensively summarized in Table 1. No patient had a documented family history of neurogenic tumors or clinically significant pre-existing comorbidities.

**Table 1:** Demographics and characteristics of RGN in all 51 patients.

Variables	*N*	% or range
Total	51	(100)
Gender (male)	18	(35.3)
Median age at diagnosis (years) (range)	28	(12–73)
Chief complains		
Physical examinations	29	(56.9)
Abdominal discomfort	16	(31.4)
Lumbago	3	(5.8)
Others (hematuria, chest tightness, lower limb pain)	3	(5.8)
Nutrition index (mean ± SD)		
Haemoglobin (g/L)		(133 ± 5.8)
Albumin (g/L)		(39.3 ± 2.4)
BMI (kg/m^2^)		(21.4 ± 3.9)

### 3.2 Preoperative labs, images and biopsies

**Figure 1:**
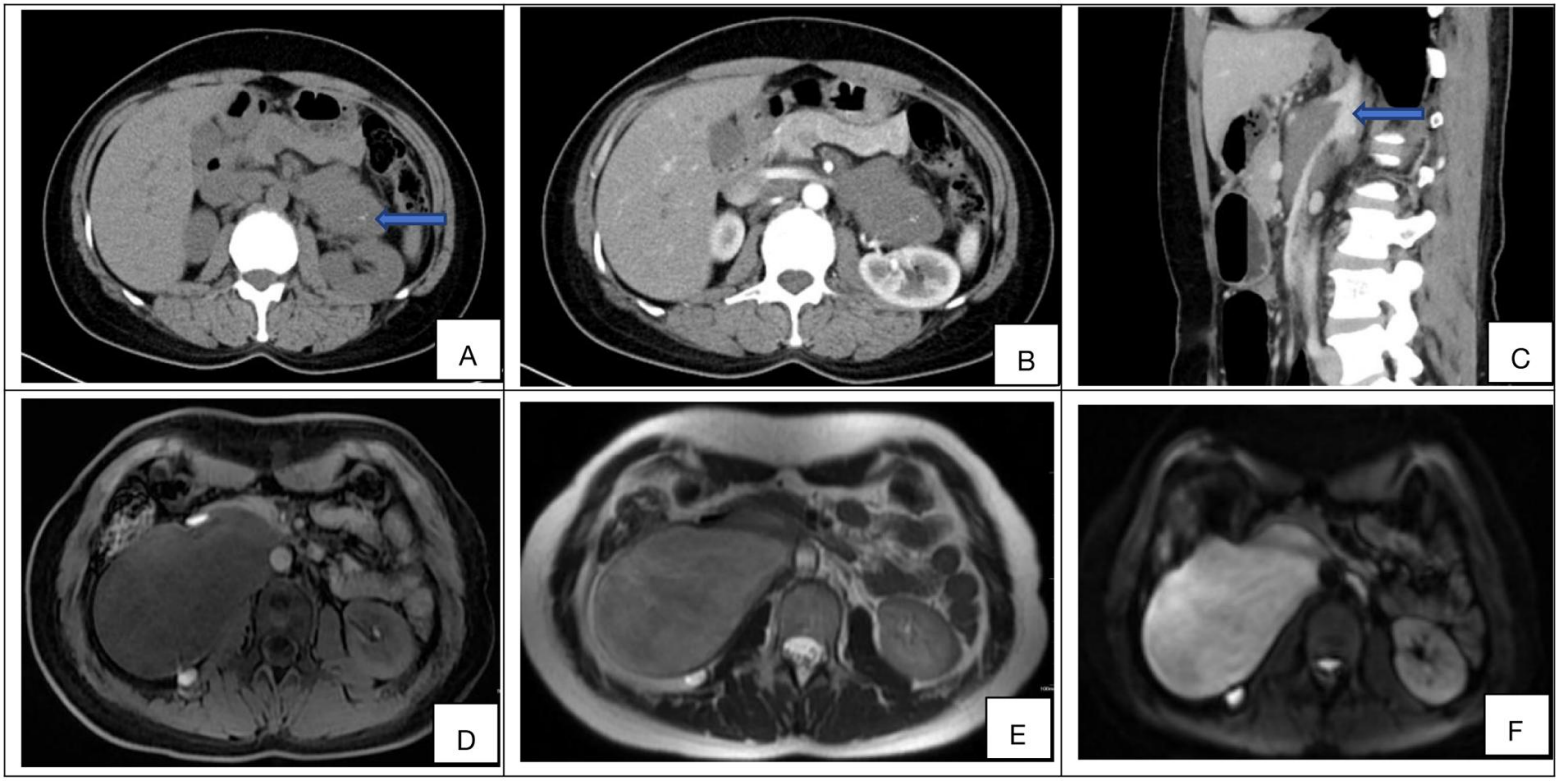
**(A–F)** Representative preoperative imaging findings of retroperitoneal ganglioneuroma. **(A)** Axial non-contrast CT demonstrates a hypoattenuating mass (mean density: 32.63 HU) with internal curvilinear calcifications (blue arrow). **(B)** Axial contrast-enhanced CT (portal venous phase) shows minimal intralesional enhancement (ΔHU = +6.85) relative to muscle. **(C)** Sagittal non-contrast CT reveals circumferential encasement of the celiac trunk and superior mesenteric artery without luminal stenosis (blue arrow). **(D)** Axial T1-weighted MRI (non-contrast) displays low signal. **(E)** Axial T2-weighted MRI demonstrates heterogeneous hyperintensity with internal septations. **(F)** Diffusion-weighted imaging (DWI) shows high intensity, and the compared sequences show varying degrees of enhancement (from none to strong).

Preoperative laboratory assessment included complete blood count (CBC), comprehensive metabolic panel, and tumor marker evaluation. Median hemoglobin was 132 g/L and albumin 39.2 g/L. All patients exhibited normal serum levels of tumor markers, including α-fetoprotein (AFP), carbohydrate antigen 19-9 (CA19-9), cancer antigen 125 (CA-125), and carcinoembryonic antigen (CEA).Of the 51 patients, catecholamine and vasoactive intestinal peptide testing was performed in 13, all of whom had negative results.All 51 patients underwent contrast-enhanced computed tomography (CT), with six (11.8%, 6/51) receiving supplemental contrast-enhanced magnetic resonance imaging (MRI). Characteristic CT features included well-circumscribed hypoattenuating masses demonstrating minimal contrast enhancement (median enhancement: 35.08 HU). Perivascular encasement of major abdominal vessels was frequently observed, with thin peripheral calcifications present in some cases (Figures 1A–C). On MRI, lesions demonstrated homogeneous T1 hypointensity. T2-weighted imaging revealed iso- to hyperintense signal with inverse proportionality to myxoid stroma content: intermediate-high signal correlated with predominant cellular components/collagen fibers, while marked hyperintensity indicated abundant myxoid stroma. Diffusion-weighted imaging showed hyperintensity, with post-contrast enhancement varying from absent to intense (Figures 1D–F). The radiological methods and corresponding findings are summarized in Table 2. Preoperative core needle biopsy was performed in 18 patients (35.3%), yielding histopathological diagnoses of neurogenic tumor (*n* = 6, 33.3%) and definitive ganglioneuroma (*n* = 12, 66.7%).

**Table 2:** The radiological methods and corresponding findings of RGN in all 51 patients.

Variables	*N*	% or range
CT	51	(100)
Benign neurogenic tumors	42	(82.4)
Ganglioneuroma	32	(62.7)
Neurofibroma	4	(7.8)
Schwannoma	4	(7.8)
Benign paraganglioma	2	(3.9)
Malignant tumors	6	(11.8)
Liposarcoma	4	(7.8)
Leiomyosarcoma	2	(3.9)
Other diagnoses	3	(5.9)
Lymphangioma	2	(3.9)
pheochromocytoma	1	(2.0)
Calcification	12	(23.5)
MRI	6	(11.8)
Benign neurogenic tumors	5	(83.3)
Ganglioneuroma	4	(66.7)
Neurofibroma	1	(16.7)
Malignant tumors	1	(16.7)
Leiomyosarcoma	1	(16.7)
Tumor location		
Left retroperitoneal	21	(41.2)
Right retroperitoneal	30	(58.8)
Envelops major vasculature	34	(66.7)
Abdominal aorta	10	(19.6)
Superior mesenteric artery	9	(17.6)
Inferior vena cava	6	(11.8)
Renal vessels	6	(11.8)
Iliac vessels	3	(5.9)

### 3.3 Surgical details

Surgical management was guided by a dedicated multidisciplinary team (RPT-MDT) specializing in retroperitoneal tumors, including surgical oncology, radiology, and pathology. All procedures were performed by an experienced retroperitoneal oncology surgical team. Approach selection was individualized based on tumor topography and size: 41 patients (80.4%) underwent open resection, while 10 (19.6%) had laparoscopic surgery. Maximum tumor diameter ranged from 4.1 to 30.2 cm (mean ± SD: 13.06 ± 5.77 cm). Mean operative duration was 259.0 ± 153.1 min. Intraoperative blood loss exhibited substantial variation (range: 50–4,500 ml; median: 450 ml; IQR: 200–950 ml). 45 and 6 patients underwent R0/R1 and R2 resection, respectively. 14 patients underwent multivisceral resection during surgery.Postoperative complications occurred in 11 patients (21.6%, Clavien-Dindo Grade I/II: *n* = 10; Grade V: *n* = 1), including:

*Gastroparesis requiring prokinetics (*n* = 3)*
*Biochemically-diagnosed pancreatic fistula (*n* = 3)*
*Transaminitis (*n* = 2)*
*Superficial wound infections (*n* = 2)*

One mortality occurred on postoperative day 8 secondary to bowel ischemia with septic shock. The remaining patients achieved clinical recovery with conservative management. Median hospital stay was 10.2 days (IQR: 8–14 days). Comprehensive surgical outcomes including R0/R1 resection status are detailed in Table 3.

**Table 3:** Operative outcome of RGN in all 51 patients.

Variables	*N*	% or range
Total	51	(100)
Surgical approaches		
Traditional open resection	41	(80.4)
Laparoscopic surgery	10	(19.6)
OPT (min)		(259.0 ± 153.1)
IBL (mL)		(50–4,500)
Resection margin		
R0/R1	45	(88.2)
R2	6	(11.8)
Combined resection of organs	14	(27.5)
Diaphragm	6	(11.8)
Adrenal	3	(5.9)
Spleen	2	(3.9)
Pancreas	2	(3.9)
Renal	1	(2.0)
Complication		
Total	11	(21.6)
Clavien-Dindo Grade I/II	10	(90.9)
Grade V	1	(9.1)
Gastroparesis	3	(5.9)
Pancreatic fistula	3	(27.3)
Transaminitis	2	(3.9)
Wound infections	2	(3.9)
Mortality	1	(2.0)

### 3.4 Histopathological

**Figure 2:**
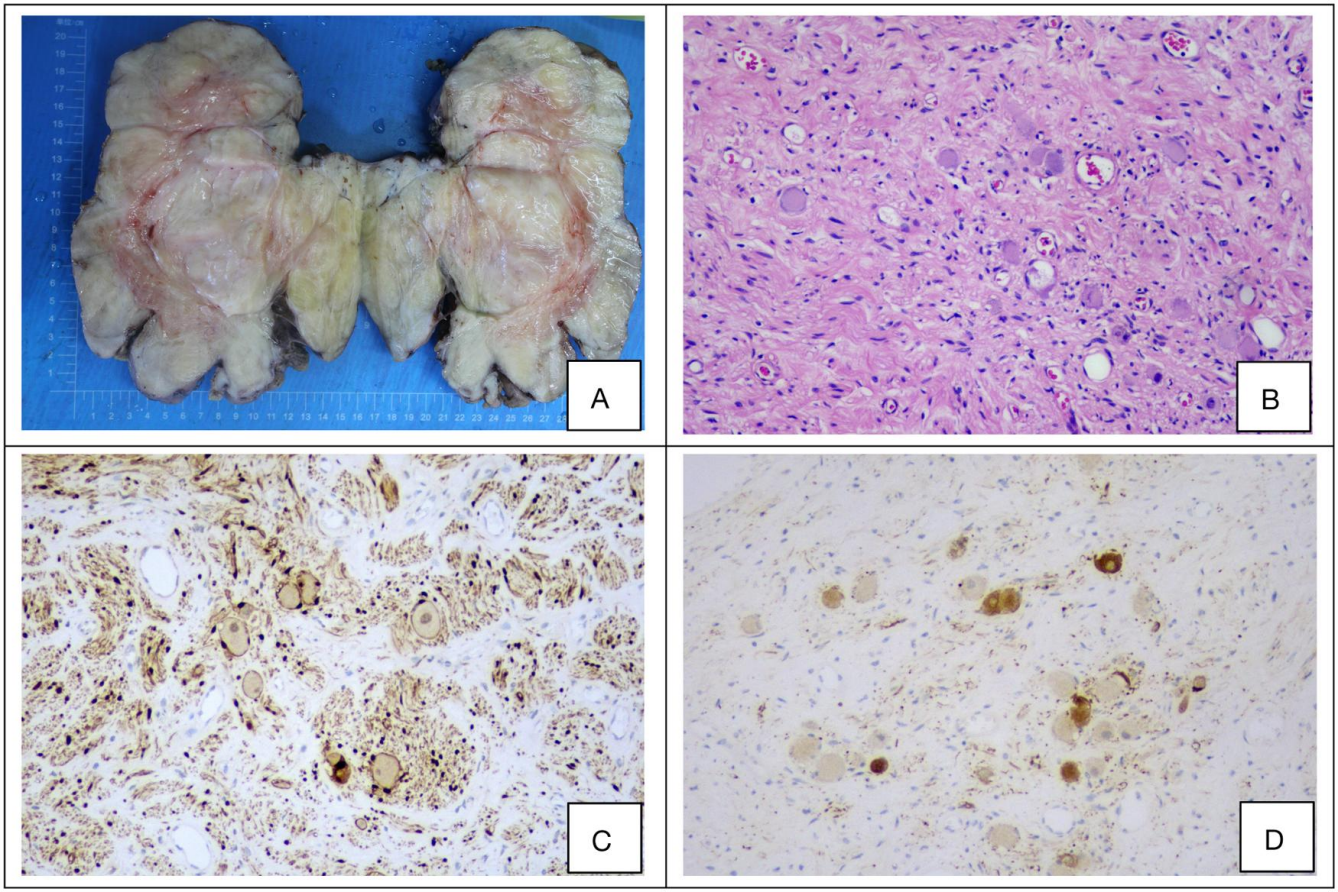
**(A–D)** Representative histopathological and immunohistochemical features of a retroperitoneal ganglioneuroma. **(A)** Gross appearance of the resected tumor specimen. **(B)** Hematoxylin and eosin (H&E) staining reveals fascicles of spindle cells and scattered mature ganglion cells (original magnification ×100). **(C,D)** Immunohistochemical staining demonstrates strong positivity for S-100 and neuron-specific enolase (NSE) (original magnification ×100 for both).

The diagnosis of ganglioneuroma (GN) is established based on characteristic histopathological and immunohistochemical findings. Macroscopically, the tumors typically appear as round, oval, or lobulated masses with well-defined borders and a complete or partial capsule. The cut surface is firm and gray-white to gray-yellow in color, often displaying a distinctive whorled or fasciculated architecture, and is generally devoid of hemorrhage, necrosis, or cystic change (Figure 2A). Microscopic examination reveals a biphasic morphology composed of fascicles of spindle-shaped Schwann cells interspersed with mature ganglion cells, set within a myxoid stromal background (Figure 2B). Immunohistochemically, the neoplastic cells consistently expressed S-100 and NSE, with positivity rates of 100% and 92.2%, respectively (Figures 2C,D). The clinicopathological characteristics and immunohistochemical profiles of the 51 retroperitoneal ganglioneuroma cases are summarized in Table 4.

**Table 4:** Clinicopathologic characteristics and immunohistochemical profiles of the 51 patients with retroperitoneal ganglioneuroma.

Tumor features	*N*	% or range
Tumor size		(4.1–30.2 cm)
<5cm	5	(9.8)
5–10 cm	24	(47.1)
≥10 cm	22	(43.1)
Tumor shape		
Regular	23	(45.1)
Irregular	28	(54.9)
Tumor border		
Clear	42	(82.4)
Not clear	9	(17.6)
Tumor texture		
Tough	34	(66.7)
Soft	11	(21.6)
Hard	6	(11.8)
Tumor degeneration		
Hemorrhage	2	(3.9)
Calcification	1	(2.0)
Necrosis	0	(0)
Cystic degeneration	0	(0)
Antigen positive		
S-100	51	(100)
NSE	47	(92.2)

### 3.5 Follow-up results

No adjuvant therapy (including hormonal, radiotherapeutic, or chemotherapeutic interventions) was administered postoperatively. Patients underwent scheduled follow-up via structured telephone interviews and clinical evaluations at 3-month intervals for the first two years, then biannually thereafter. Five patients (9.8%) were lost to follow-up, yielding a 90.2% completion rate. The median follow-up duration was 62 months (range 6–120). Among six patients with macroscopic residual disease (R2 resection), serial imaging demonstrated stable disease without radiological progression (defined as <20% increase in maximum diameter) (8). Crucially, no disease recurrence was observed in any patient with complete follow-up. There were no disease-specific mortalities during the study period. Given the absence of endpoint events (recurrence or disease-related death), formal time-to-event analysis was precluded.

## 4 Discussion

Ganglioneuroma (GN), a rare benign neoplasm of neural crest origin, has an estimated incidence of approximately 1 per million population. RGN accounts for 0.72%–1.6% of primary retroperitoneal tumors, predominantly affecting children and young-to-middle-aged individuals (<40 years) (3). Consistent with previous epidemiological data, the majority of our cohort (74.5%, 38/51) was aged <40 years, with a female predominance (64.7%, 33/51) (9). Notably, 56.9% (29/51) of patients were asymptomatic and diagnosed incidentally during unrelated imaging evaluations, highlighting the tumor's indolent biology. This finding underscores the critical role of cross-sectional imaging (contrast-enhanced CT/MRI) in detection, as nonspecific symptoms—including abdominal discomfort (31.4%, 16/51) and low back pain (5.9%, 3/51)—frequently delay diagnosis. Although catecholamine or vasoactive intestinal peptide secretion has been documented in rare cases (potentially causing hypertension/diarrhea), no paraneoplastic syndromes manifested in our cohort (10). Despite typically indolent behavior, the retroperitoneal location and local expansive potential of GN present unique diagnostic and therapeutic challenges (11).

RGN pose significant diagnostic challenges due to nonspecific clinical presentations and imaging overlap with malignant neoplasms. In our cohort, 56.9% (29/51) of cases were incidentally detected, underscoring the critical role of advanced imaging in early diagnosis. Comprehensive radiologic evaluation facilitates precise tumor localization, characterization, and surgical planning; however, meticulous differentiation from other retroperitoneal tumors remains imperative. Key computed tomography (CT) features include well-circumscribed oval, crescentic, or lobulated masses exhibiting homogeneous or mildly heterogeneous hypoattenuation on non-contrast studies, with characteristic hypoenhancing lesions on contrast-enhanced CT (performed universally) (12). Discrete punctate calcifications were observed in 20% (10/51) of cases. Magnetic resonance imaging (MRI) revealed homogeneous hypointensity on T1-weighted imaging (T1WI), while T2-weighted imaging (T2WI) demonstrated iso- to hyperintensity—where signal intensity inversely correlated with myxoid stroma content—manifesting as intermediate-high signal in cellular/collagen-rich regions vs. marked hyperintensity in myxoid-dominant areas (13, 14). Diffusion-weighted imaging (DWI) showed hyperintensity, and post-contrast sequences exhibited variable enhancement (absent to intense). Calcification morphology provides critical diagnostic value: well-defined punctate calcifications in GNs contrast sharply with the amorphous, coarse calcifications typical of neuroblastomas, enabling differentiation from malignant neurogenic tumors (e.g., ganglioneuroblastoma, neuroblastoma) (15). These features align with established literature and aid distinction from other soft-tissue neoplasms.

Preoperative core needle biopsy—performed in 35.3% [18/51] of patients—proved essential for discriminating benign GNs from malignant counterparts (16). Notably, 33.3% (6/18) of preoperative biopsies yielded nonspecific “neurogenic tumour” diagnoses, requiring supplementary immunohistochemical staining—typically positive for S-100 and NSE, and negative for desmin, smooth muscle actin, and CD34—for definitive classification (17). Histologically, GN is defined by the absence of mitotic activity, intermediate cells, neuroblasts, and necrosis, which are essential criteria for differentiating it from ganglioneuroblastoma and paraganglioma according to the WHO classification (18). The diagnostic accuracy of biopsy is limited by sampling heterogeneity and anatomic constraints. The inherent risk of sampling error in core needle biopsy may miss foci of ganglioneuroblastoma or malignant transformation, potentially leading to false-negative diagnoses and inappropriate management (19). Furthermore, the retroperitoneal location of many tumors, often adjacent to major vessels such as the aorta and vena cava, entails non-negligible risks of vascular injury, hemorrhage, or pseudoaneurysm. Given these limitations, surgical excision was regarded as the optimal approach in resectable cases. It provides the entire specimen for definitive histopathological evaluation—the diagnostic gold standard—while simultaneously achieving curative intent, thereby circumventing the risks and potential delays of a two-stage diagnostic-surgical pathway. In our cohort, preoperative biopsy was generally reserved for patients with initially unresectable disease or those in whom imaging suggested alternative pathologies such as lymphoma or sarcoma, which would necessitate fundamentally different treatment strategies.

Surgical resection constitutes the definitive management for retroperitoneal ganglioneuroma, with our specialized multidisciplinary team (RPT-MDT) achieving R0/R1 resection in 88.2% (45/51) of cases. This outcome reflects the technical expertise of high-volume tertiary centers where all procedures were performed by dedicated retroperitoneal oncology surgeons. Individualized approach selection—open resection in 80.4% (41/51) vs. laparoscopic in 19.6% (10/51)—was guided by tumor topography and size (mean diameter: 13.06 ± 5.77 cm), with minimally invasive techniques reserved for anatomically favorable cases. Nevertheless, significant challenges persisted: 21.6% (11/51) experienced major morbidities (Clavien-Dindo I/II: *n* = 10; Grade V: *n* = 1) including gastroparesis requiring prokinetics (*n* = 3), biochemically-diagnosed pancreatic fistula (*n* = 3), and hepatic injuries (*n* = 2), directly attributable to tumor proximity to critical structures. One mortality (1.9%) occurred on postoperative day 8 secondary to SMA injury-induced bowel ischemia, while vascular encasement necessitated R2 resection in 11.8% (6/51) despite meticulous dissection along tumor pseudocapsules and 5-0 polypropylene suture repair of vascular injuries.Technical refinement was essential for tumors encasing major vessels, particularly the thin-walled superior mesenteric artery (SMA) where repair carried high stenosis risk. Preoperative vascular quantification informed our dissection strategy commencing at the aortic bifurcation. For circumferential involvement, internal tumor incision identified dissection planes, while SMA injuries mandated immediate tumor resection followed by patency-focused repair. When stenosis compromised bowel viability (assessed intraoperatively), oblique incision maximizes the diameter of the anastomosis and implants an artificial blood vessel at the defect site to prevent tension-induced vasospasm.These outcomes underscore three imperatives: First, RPT-MDT collaboration (surgical oncology, radiology, pathology) enables preoperative risk stratification and intraoperative decision-making during complex dissections. Second, advanced vascular expertise is non-negotiable for hemorrhage control during multivisceral resections (*n* = 14) and SMA reconstruction. Third, judicious patient selection for laparoscopy must weigh tumor size against vascular involvement patterns, as inappropriate minimally invasive attempts risk catastrophic complications in this anatomically complex territory (13, 17). Combined multi-organ resection was performed in fourteen patients; however, the consequent functional sequelae and quality of life were not quantified. Future prospective studies should incorporate patient-reported outcome measures (PROMs) to address this critical aspect.Moreover, this study originates from a high-volume sarcoma referral center, and as such, our patient cohort may be biased towards more complex cases with larger tumors or more challenging anatomical relationships. Therefore, the rates of multivisceral resection and complications reported here may not be directly generalizable to all clinical settings.

With a median follow-up of 62 months (IQR: 36–108), no instances of disease recurrence, distant metastasis, or treatment-related mortality were observed in our cohort, supporting the characteristically indolent clinical behavior of ganglioneuroma (GN). Notably, none of the patients who underwent R2 resection (11.8%) experienced tumor progression, reinforcing the role of subtotal resection as a viable alternative when complete excision is precluded by critical vascular involvement (10, 20). This strategy, however, necessitates careful risk–benefit evaluation, particularly in younger patients, given the documented—albeit rare—potential for malignant transformation to ganglioneuroblastoma (7). Our outcomes align with previously published series. In a study of 32 GN patients, no recurrences were reported among 24 surgically treated cases during a mean follow-up of 15.8 months. Of the six patients with residual tumors, none showed progression, and two even exhibited regression on subsequent imaging. Similarly, two patients managed with active surveillance maintained stable, asymptomatic disease over several years (3). In contrast, a Tunisian cohort documented an early local recurrence four months postoperatively, attributed to residual tumor; nevertheless, the lesion subsequently stabilized on serial CT, further corroborating the generally indolent course of GN even in the setting of recurrence (13). Collectively, these findings advocate for an individualized management approach. Surgical resection remains the cornerstone of treatment for patients with significant tumor burden or diagnostic uncertainty. Principal indications for intervention include: (1) the risk of future mass effect from larger tumors (e.g., >5 cm) on critical adjacent structures such as the great vessels, ureters, or spinal cord; (2) the limited ability of preoperative imaging to reliably exclude malignancy, particularly in lesions with atypical radiological features; and (3) patient preference, as some individuals, despite comprehensive counseling, opt for resection to alleviate anxiety. Conversely, active surveillance represents a reasonable alternative for selected patients—particularly those with small, asymptomatic, radiographically typical tumors, or those with elevated operative risk.This study has several limitations. Although the median follow-up of 62 months is substantial, it may still be inadequate to detect very late events given the indolent natural history of ganglioneuroma. The retrospective, single-center design and limited cohort size may also introduce selection bias. Furthermore, the exclusion of patients with recurrent disease may have resulted in a cohort with a more favorable prognosis, potentially leading to overestimation of the recurrence-free survival rates reported herein. Future multicenter studies with extended follow-up are warranted to validate these findings, identify predictors of surgical morbidity, and clarify the true incidence of late recurrence or malignant transformation, particularly after incomplete resection.

## 5 Conclusion

In summary, retroperitoneal ganglioneuroma is a rare benign tumor with an excellent prognosis, most frequently found in children and young adults. Although often asymptomatic, it can cause nonspecific mass effects or, less commonly, functional symptoms. Radiological imaging is the mainstay of preoperative diagnosis, whereas pathology provides definitive confirmation. Management should be individualized: whereas surgery offers excellent outcomes, active surveillance is a valid option for select patients. Further research is needed to refine treatment strategies.

## Data Availability

The raw data supporting the conclusions of this article will be made available by the authors, without undue reservation.
